# Can Kushen injection combined with TACE improve therapeutic efficacy and safety in patients with advanced HCC? a systematic review and network meta-analysis

**DOI:** 10.18632/oncotarget.20921

**Published:** 2017-09-15

**Authors:** Yingshi Zhang, Fuhai Hui, Yue Yang, Haixiao Chu, Xiaochun Qin, Mingyi Zhao, Qingchun Zhao

**Affiliations:** ^1^ Department of Clinical Pharmacy, Shenyang Pharmaceutical University, Shenyang 110016, P.R. China; ^2^ Department of Pharmacy, General Hospital of Shenyang Military Area Command, Shenyang 110840, P.R. China

**Keywords:** transarterial chemoembolization, TACE, compound Kushen injection, advanced hepatocellular carcinoma, network meta-analysis

## Abstract

**Objective:**

To assess the comparative efficacy and safety of combination treatment with Compound Kushen Injection (CKI) and transarterial chemoembolization (TACE) in patients with advanced hepatocellular carcinoma (HCC) through a systematic review and network meta-analysis and to identify the best conditions for using CKI.

**Materials and Methods:**

We performed a network meta-analysis based on randomized controlled trials. We searched databases for studies published by August 2017. The prespecified primary efficacy outcome was treatment response, while the secondary efficacy outcomes were KPS score, Child-Pugh score, overall survival rate, clinical symptoms, and improvements in immune function and liver function; we performed subgroup analyses and meta-regressions according to the different TACE arms, CKI dosage, composition of CKI, embolizing agents and treatment duration. The safety outcomes were side effects. We conducted pairwise meta-analyses using a random-effects model and then performed random-effects network meta-analyses.

**Results:**

A total of 44 trials, involving 3778 patients and 22 intervention arms, were eligible. TACE+CKI could significantly increase treatment response (1.85, 1.56 to 2.20) and improve therapeutic efficacy based on the secondary outcomes. Significant efficacy was observed in most subgroups. Network meta-analysis revealed that CKI was very suitable for combination treatment when the TACE arm included 5-fluorouracil+epirubicin+hydroxycamptothecin, pirarubicin+hydroxycamptothecin and 5-fluorouracil+pirarubicin+mitomycin+hydroxycamptothecin. The study is registered with PROSPERO (CRD42017073181).

**Conclusions:**

Regarding efficacy, TACE+CKI offers clear advantages for patients with advanced HCC. Moreover, patients should be encouraged to accept CKI, especially when the chemotherapeutic drugs in TACE have high levels of adriamycins (epirubicin and pirarubicin) and hydroxycamptothecin.

## INTRODUCTION

Liver cancer (primarily hepatocellular carcinoma, HCC) is one of the most common cancers with dismal outcomes including cancer-related death [1–2]. HCC is a common disease and accounts for 54% of the total number of cancer patients worldwide, with more than 600,000 related deaths estimated each year [1]. HCC is the fifth-most common malignancy and the second leading cause of cancer-related death worldwide; the 5-year survival rate of HCC is 15–17% [3]; the incidence rates of liver cancer have continued to increase rapidly by approximately 3% per year in women and 4% per year in men, although the rates have begun to decline in adults younger than 50 years of age [4]. Only a small proportion of patients with early-stage HCC can benefit from radical treatment options, such as surgical resection and orthotopic liver transplantation. Although hepatic resection offers hope of cure in patients suffering from HCC, only a small proportion (10–15%) of HCC patients are eligible for this procedure [5]. However, surgical resection is not the first treatment choice for HCC patients with large lesions or poor liver function.

Transarterial chemoembolization (TACE) as a kind of palliative care and management that is prescribed for most patients with advanced HCC to prevent and relieve suffering and improve the quality of life. This is a standard and minimally invasive procedure developed for HCC patients who are not eligible for complete resection [6] and is the most widely used primary treatment for advanced HCC [7]. This procedure combines transcatheter delivery of chemotherapy emulsions with lipiodol, followed by vascular stagnation achieved with embolization agents. TACE results in partial responses in 15–55% of patients, and it significantly delays tumor progression and macrovascular invasion. The most common TACE regimens include one, two and three chemotherapeutic agents, such as doxorubicin (ADM), cisplatin (DDP), epirubicin (EPI), hydroxycamptothecin (HCPT) and mitomycin (MMC) [8–9]. However, sensitivity analysis of results from efficacy studies has shown a significant benefit of TACE with cisplatin or doxorubicin [10]. Thus, we included TACE treatment arms with or without adriamycins/platinum for subgroup analyses. However, regarding the morbidity and mortality rate due to advanced unresectable HCC, the efficacy of TACE alone is not satisfactory. In addition, TACE has its own limitations, as it can further affect liver functions and damage the hepatic arterial system. As a result, TACE is not appropriate for HCC patients with poor liver functions, particularly patients with cirrhosis [11]. Therefore, in recent years, increasing attention has been focused on the effectiveness of TACE therapy for liver cancer combined with other non-chemical drugs or palliative therapies, including Compound Kushen Injection (CKI).

CKI is a kind of traditional Chinese medicinal preparation that is widely used in clinics. The main components of Kushen injection are matrine (Sophorae Flavescentis Radix) and tufuling (Smilacis Glabrae Rhizoma) with inactive ingredients such as polysorbate 80, sodium hydroxide and acetic acid. Its functions include relieving fever, dampness and blood stasis, detoxification, dissolution of tumors and alleviating pain, among others” [12]. Matrine is the main active ingredient of Kushen injection. Moreover, matrine exhibits anti-tumor effects against breast cancer cells (MCF-7), gastric cancer cells (SGC-7901 and MKN45), gallbladder cancer cells (GBC-SD) and osteosarcoma cells (UMR-108), and can also significantly inhibit the proliferation of HCC cells via a mechanism that may be related to the induction of apoptosis [13]. Therefore, we speculate that CKI can be used in combination with TACE in HCC treatment to relieve the clinical symptoms of cancer, reduce the side effects of chemotherapy, improve the quality of life and prolong the survival of patients. However, there is a lack of high-quality evidence to support this notion. To further explore these issues and to identify the efficacy and safety of combination treatment with CKI and TACE, we performed a network meta-analysis of all available randomized controlled trials on patients with advanced HCC. Until now, no other review [14–15] has provided a comprehensive overview of this subject using meta-regressions and network meta-analyses.

## RESULTS

### Description of the network and patients

**Figure 1:**
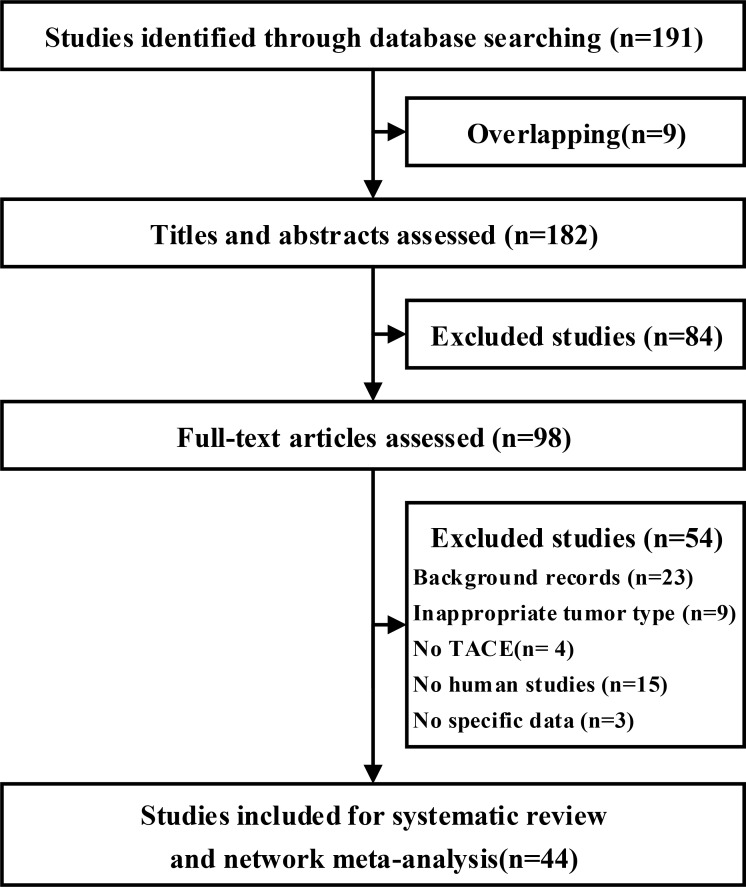
Flowchart of study selection for the systematic review and network meta-analysis TACE, Transarterial chemoembolization.

In total, 191 publications were retrieved from databases; after removing duplicates, 182 publications were screened based on the title and abstract, and 84 were excluded from further analysis. A total of 98 publications were included for full-text analysis. The network comprised 44 trials with 3778 patients, which were included in the standard meta-analysis [16–59]. There were 22 different TACE arms: 5-fluorouracil (5-FU)+doxorubicin (ADM)+hydroxycamptothecin (HCPT); 5-FU+Cisplatin (DDP); 5-FU+DDP+ADM; 5-FU+DDP+epirubicin (EPI); 5-FU+DDP+gemcitabine (GEM); 5-FU+DDP+HCPT; 5-FU+DDP+mitomycin (MMC); 5-FU+DDP+MMC+vincristine (VCR); 5-FU+EPI+HCPT; 5-FU+EPI+MMC; 5-FU+HCPT; 5-FU+MMC; 5-FU+MMC+pirarubicin (THP)+oxaliplatin (L-OHP); 5-FU+THP+MMC+HCPT; DDP+ADM+MMC; EPI; EPI+THP+L-OHP; L-OHP+GEM; THP; THP+camptothecin (CPT); THP+HCPT; and THP+MMC+HCPT. Figure 1 shows the flowchart representing the steps to screen for the relevant studies.

Table 1 summarizes the numbers of patients with advanced HCC according to treatment arms. Patients were grouped by different treatment arms (TACE arm and CKI arm). Table 1 also summarizes the differences in the fundamental characteristics between the two treatment arms (see complete information regarding characteristics in Supplementary Table 1). Statistical analyses showed that the two groups had similar baseline characteristics in terms of age, sex, tumor stage, and Child-Pugh score. The results of quality assessment of these studies are presented in Supplementary Figure 1, indicating that all included trials were of acceptable quality.

**Table 1:** Number of patients with advanced hepatocellular carcinoma according to study treatment

TACE arm		TACE+CKI arm	Participants	
			TACE arm	TACE+CKI arm
**Adriamycins+Platinum**	5-FU+DDP+ADM [26, 50]	15 ml/d, iv (GS) [26]	30	30
		20 ml/d, iv [50]	30	27
	EPI+THP+L-OHP [35]	20 ml/d, iv (NS or GS) [35]	20	28
	5-FU+DDP+EPI [24, 35, 41–42, 45]	15 ml/d, iv (NS) [24]	104	107
		20 ml/d, iv [35]	35	39
		20 ml/d, iv (NS or GS) [41–42]	66	66
		0.6 g/d, iv (GS) [45]	38	38
	DDP+ADM+MMC [55, 59]	150 mg/d, iv (GS) [55]	33	35
		16 ml/d, iv [59]	20	26
	5-FU+MMC+THP+L-OHP [29]	0.3 g, emulsion (matrine) [29]	24	24
**Adriamycins**	5-FU+EPI+MMC [21, 37, 40, 53]	20 ml/d, iv (NS or GS) [21, 37]	63	67
		20 ml/d, iv (NS) [40]	30	30
		20 ml/d, iv (GS) [53]	40	46
	5-FU+EPI+HCPT [19, 28]	15 ml/d, iv (NS) [19]	60	60
		20 ml/d, iv [28]	42	42
	EPI [31]	20 ml/d, iv (NS or GS) [31]	30	30
	THP+HCPT [32, 41]	20 ml/d, iv (NS or GS) [32]	30	30
		20 ml/d, iv [41]	53	53
	5-FU+ADM+HCPT [38, 44]	20 ml/d, iv (GS) [38]	31	36
		20 ml/d, iv (NS or GS) [44]	48	48
	5-FU+THP+MMC+HCPT [39]	1.2 g/d, iv [39]	30	30
	THP [48]	20 ml/d, iv [48]	20	20
	THP+CPT [20]	20 ml/d, iv (NS) [20]	42	38
	THP+MMC+HCPT [54]	150 mg/d, iv [54]	60	62
**Platinum**	L-OHP+GEM [16]	150 mg/d, iv [16]	108	108
	5-FU+DDP+MMC [17, 23, 27, 33, 46, 47]	20 ml/d, iv (NS) [17, 23, 46, 47]	141	143
		20 ml/d, iv (NS or GS) [33]	30	30
		Unknown [27]	101	98
	5-FU+DDP [22, 36]	20 ml/d, iv (GS) [22, 36]	61	61
	5-FU+DDP+GEM [30]	600 mg, iv [30]	20	20
	5-FU+DDP+MMC+VCR [49]	20 ml/d, iv (NS) [49]	25	38
	5-FU+DDP+HCPT [56]	150 mg/d, iv [56]	32	30
**Other**	5-FU+HCPT [51–52]	20 ml/d, iv (GS) [51–52]	60	60
	5-FU+MMC [57]	0.6 g, emulsion [57]	35	40
	Unknown [18, 25]	20 ml/d, iv (NS) [18]	38	38
		20 ml/d, emulsion [25]	36	34
**Baseline characteristics**	**Characteristics of patients**	**TACE arm vs TACE+CKI arm (OR, 95% CI)**		**Heterogeneity (*P*, I**^2^**)**
	**Age (year)**	-0.73 (-1.84, 0.38)^*^		0.000, 98.1%
	**Male**	1.06 (0.87, 1.30)		0.990, 0.0%
	**Tumor stage (I–II/III–IV)**	1.40 (0.93, 2.10)		0.069, 42.0%
	**Child-Pugh (A/B–C)**	1.00 (0.76, 1.33)		0.990, 0.0%

^*^Standardized mean difference; 5-FU, 5-Fluorouracil; ADM, Doxorubicin; CBP, Carboplatin; CPT, Camptothecin; DDP, Cisplatin; EPI, Epirubicin; GEM, Gemcitabine; HCPT, Hydroxycamptothecin; L-OHP, Oxaliplatin; MMC, Mitomycin; TACE, Transarterial chemoembolization; THP, Pirarubicin; VCR, Vincristine.

### Primary efficacy outcomes - treatment responses

#### Standardized meta-analysis

**Figure 2:**
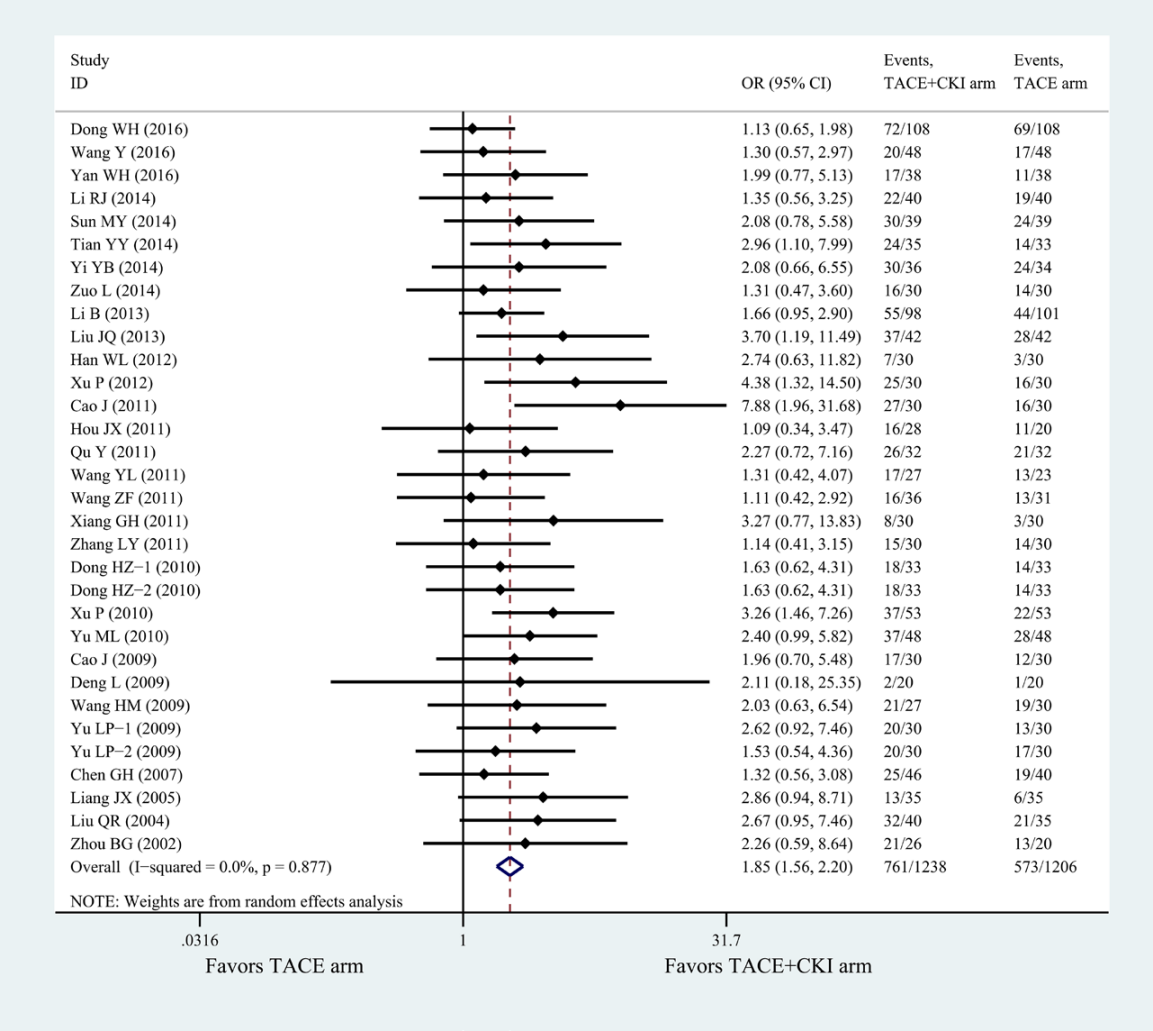
Overall efficacy of TACE+CKI vs TACE on treatment responses CKI, Compound Kushen Injection; TACE, Transarterial chemoembolization.

Figure 2 and Table 2 summarize the results of treatment responses from 32 trials (2444 patients) in patients with advanced HCC. Compared with TACE alone, treatment with TACE+CKI conferred significant therapeutic advantages (odds ratios: 1.85, 95% confidence intervals: 1.56 to 2.20) with low heterogeneity (*P* = 0.877, *I*^2^ = 0.0%; Figure 2, Table 2). Moreover, some degree of bias was observed using Begg’s test (*P =* 0.128) and Egger’s test (*P* = 0.008), and the data were of high quality according to the GRADE assessment (Table 2).

**Table 2:** Subgroup analyses, meta-regression and quality of evidence regarding treatment response rates

Subgroups	Participants (T/C)	OR (95% CI)	Heterogeneity (*P*, *I*^2^)	Meta-regression (*P*)	Quality of evidence	Publication bias	
						Begg’s (*P*)	Egger’s (*P*)
**Total (*n =* 32)**	1238/1206	1.85 (1.56, 2.20)^#^	*P* = 0.877, *I*^2^ = 0.0%		High	*P* = 0.128	*P* = 0.008^*^
TACE arms							
Adriamycins+Platinum (***n =*** 7)	212/201	1.71 (1.13, 2.57)^#^	*P* = 0.926, *I*^2^ = 0.0%	*P* = 0.113	High	*P* = 0.652	*P* = 0.443
Adriamycins (***n =*** 12)	432/417	1.97 (1.45, 2.66)^#^	*P* = 0.547, *I*^2^ = 0.0%		High	*P* = 0.63	*P* = 0.497
Platinum (***n =*** 8)	420/421	1.80 (1.29, 2.51)^#^	*P* = 0.268, *I*^2^ = 20.4%		High	*P* = 0.035	*P* = 0.017^*^
**CKI dosage**							
20 ml/d (*n =* 27)	1077/1056	1.80 (1.50, 2.17)^#^	*P* = 0.797, *I*^2^ = 0.0%	*P* = 0.845	High	*P* = 0.045^*^	*P* = 0.025^*^
< 20 ml/d (*n =* 5)	161/150	2.25 (1.35, 3.78)^#^	*P* = 0.801, *I*^2^ = 0.0%		High	*P* = 0.142	*P* = 0.400
**Composition of CKI**							
Injection (NS or GS) (*n =* 11)	453/435	1.68 (1.23, 2.29)^#^	*P* = 0.325, *I*^2^ = 12.6%	*P* = 0.753	High	*P* = 0.052	*P* = 0.025^*^
Injection (NS) (*n =* 5)	181/179	1.73 (1.13, 2.65)^#^	*P* = 0.669, *I*^2^ = 0.0%		High	*P* = 1.000	*P* = 0.497
Injection (GS) (*n =* 5)	167/162	1.80 (1.13, 2.86)^#^	*P* = 0.775, *I*^2^ = 0.0%		High	*P* = 0.312	*P* = 0.344
**Embolizing agents**							
Lipiodol (*n =* 27)	1044/1023	1.76 (1.46, 2.12)^#^	*P* = 0.813, *I*^2^ = 0.0%	*P* = 0.947	High	*P* = 0.012^*^	*P* = 0.019^*^
Lipiodol+Gelfoam (*n =* 4)	895/1860	2.69 (1.58, 4.57)^#^	*P* = 0.934, *I*^2^ = 0.0%		High	*P* = 0.734	*P* = 0.832
**Duration**							
≤ 10 d (*n =* 3)	165/168	1.37 (0.85, 2.21)	*P* = 0.418, *I*^2^ = 0.0%	*P* = 0.353	High	*P* = 0.115	*P* = 0.286
10–30 d (*n =* 15)	489/477	2.06 (1.56, 2.72)^#^	*P* = 0.622, *I*^2^ = 0.0%		High	*P* = 0.296	*P* = 0.010^*^
> 30 d (*n =* 12)	510/489	1.82 (1.40, 2.36)^#^	*P* = 0.841, *I*^2^ = 0.0%		High	*P* = 0.234	*P* = 0.348

^#^Results with significant differences; ^*^Publication bias. CKI, Compound Kushen Injection; TACE, Transarterial chemoembolization.

Subgroup analyses and meta-regressions were used to explore the source of heterogeneity between the TACE+CKI and TACE arms regarding therapeutic efficacy (Table 2). The meta-regression results showed that the different TACE arms might have influenced the final results with a *P* value of 0.113. Significant efficacy was observed in all subgroups except for the group with the treatment duration of fewer than 10 days. Low degree of heterogeneity was observed in all subgroups with strong significance. In general, meta-regression did not reveal the source of heterogeneity in our pairwise meta-analysis; however, in all subgroups of TACE+CKI vs TACE alone, positive results were observed with the combination treatment. Although meta-regression results revealed differences between the TACE arms compared to the other grouping methods (Table 2), the *P* values were not significant. Therefore, we need to perform further comparisons of differences in TACE arms by network meta-analysis.

### Network meta-analysis

**Figure 3:**
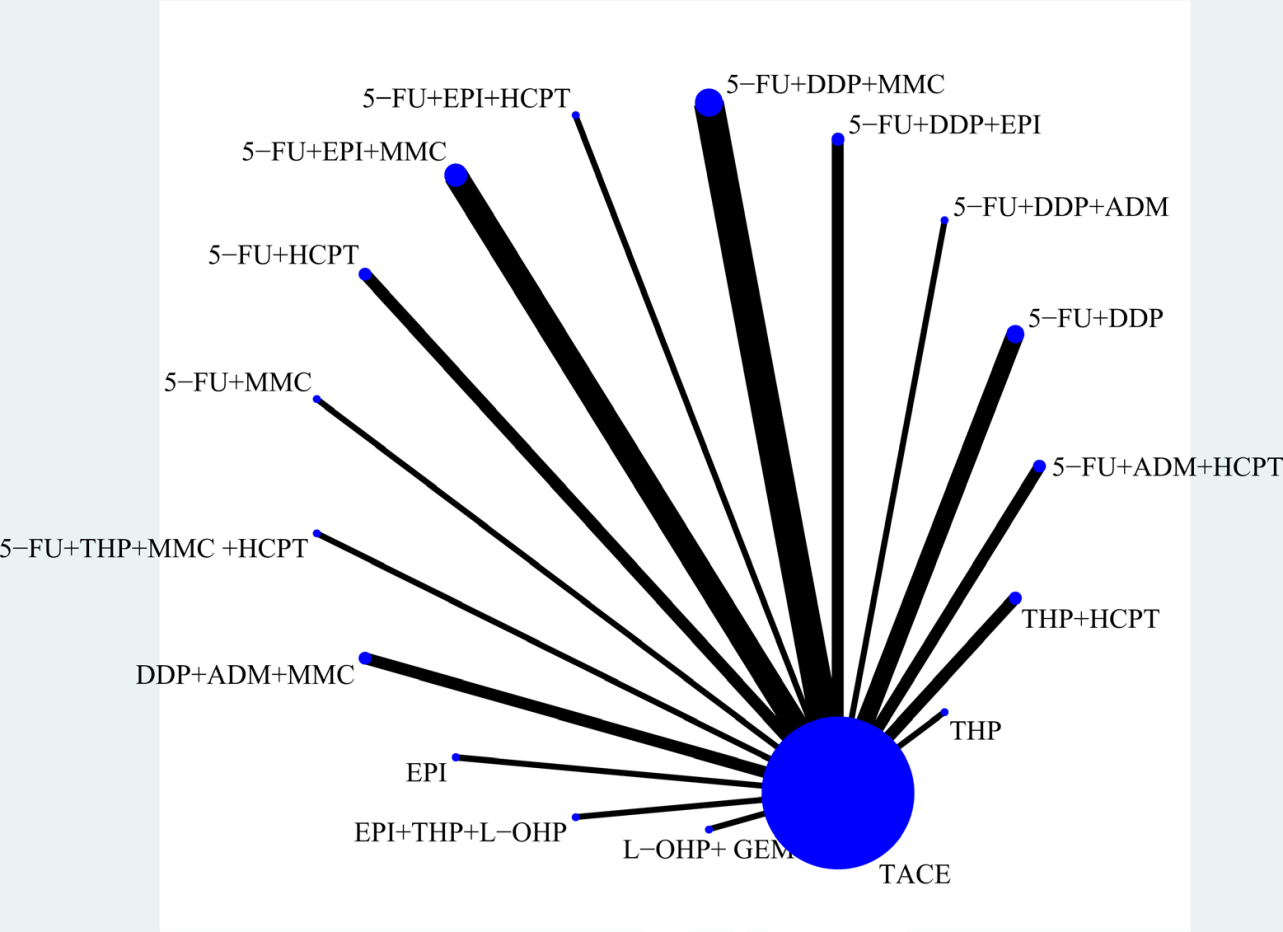
Network of eligible comparisons of efficacy of treatment The size of the nodes and the thickness of the edges are weighted according to the number of studies evaluating each treatment and direct comparison, respectively. The size of each circle is proportional to the number of randomly assigned patients and represents the sample size. The width of the lines corresponds to the number of trials. 5-FU, Fluorouracil; ADM, Doxorubicin; CBP, Carboplatin; CKI, Compound Kushen Injection; DDP, Cisplatin; EPI, Epirubicin; GEM, Gemcitabine; HCPT, Hydroxycamptothecin; L-OHP, Oxaliplatin; MMC, Mitomycin; TACE, Transarterial chemoembolization; THP, Pirarubicin; VCR, Vincristine.

Figure 3 displays the network weight of eligible comparisons of treatment efficacy along with the available, direct comparisons and network of the trials. Network meta-analysis suggested that in comparison with the TACE-only arm, 5-FU+EPI+HCPT ranked as the best (4.04, 95% credible intervals: 1.37 to 11.96), followed by THP+HCPT (3.62, 1.88 to 6.97), 5-FU+THP+MMC+HCPT (3.26, 0.86 to 12.31), 5-FU+MMC (2.91, 1.08 to 7.84), DDP+ADM+MMC (2.63, 1.15 to 6.04), EPI (2.80, 0.73 to 10.77), 5-FU+DDP (2.27, 1.09 to 4.72), 5-FU+HCPT (2.08, 1.01 to 4.29), THP (2.26, 0.30 to 17.07), 5-FU+DDP+MMC (1.95, 1.34 to 2.83), 5-FU+ADM+HCPT (1.75, 0.92 to 3.32), 5-FU+DDP+ADM (1.66, 0.79 to 3.52), 5-FU+DDP+EPI (1.68, 0.86 to 3.30), EPI+THP+L-OHP (1.29, 0.43 to 3.88), 5-FU+EPI+MMC (1.29, 0.81 to 2.07) and L-OHP+GEM (1.18, 0.68 to 2.06).

**Figure 4:**
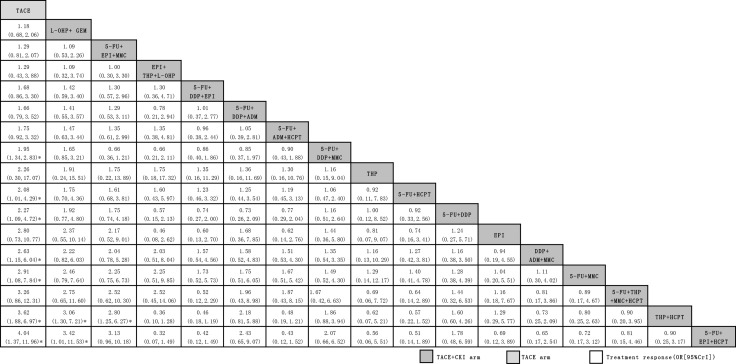
Summary of ORs and CrIs from network meta-analysis TACE arms are reported in order of the efficacy of treatment ranked according to SUCRA. Comparisons between treatments should be read from left to right. For efficacy of treatment, OR>1 indicates favorable efficacy for the indicated treatment arm compared the efficacy of the other arm. 5-FU, Fluorouracil; ADM, Doxorubicin; CBP, Carboplatin; CKI, Compound Kushen Injection; DDP, Cisplatin; EPI, Epirubicin; GEM, Gemcitabine; HCPT, Hydroxycamptothecin; L-OHP, Oxaliplatin; MMC, Mitomycin; TACE, Transarterial Chemoembolization; THP, Pirarubicin; VCR, Vincristine.

When we assessed the comparative efficacy of CKI, 5-FU+EPI+HCPT, THP+HCPT, 5-FU+MMC, DDP+ADM+MMC, 5-FU+DDP, 5-FU+HCPT and 5-FU+DDP+MMC were superior to the TACE-only arm. Treatment arms were comparable with one another in improving responses, with a significant difference found in the 5-FU+EPI+HCPT vs L-OHP+GEM groups (3.42, 1.01 to 11.53), the THP+HCPT vs L-OHP+GEM groups (3.06, 1.30 to 7.21) and the THP+HCPT vs 5-FU+DDP+EPI groups (2.80, 1.25 to 6.27) (Figure 4).

Therefore, CKI can improve the efficacy of TACE regimens with adriamycins (EPI and THP), such as 5-FU+EPI+HCPT, THP+HCPT and 5-FU+THP+MMC+HCPT. Similar results are illustrated in Table 2 with the highest OR values in subgroup analysis.

### Secondary efficacy outcomes

Table 3 summarizes the results of the secondary outcomes associated with the different TACE arms. We found that combination therapy with TACE+CKI improves the KPS score, Child-Pugh score and overall survival rate. Moreover, combination therapy with CKI can reduce the clinical symptoms, improve immune function and liver function. The differences in most of the outcomes between TACE-only and TACE-CKI groups are significant, with mostly moderate to high-quality evidence according to GRADE assessment and moderate publication bias.

**Table 3:** Secondary efficacy outcomes and quality of evidence

Outcomes	Subgroups/Subscales	Participants (T/C)	OR (95% CI)	Heterogeneity (*P*, I^2^)	Meta-regression (*P*)	Quality of evidence	Publication bias	
							Begg’s (*P*)	Egger’s (*P*)
**KPS improvement**	**Total** (*n =* **11**)	451/445	3.26 (2.42, 4.39)^#^	*P* = 0.533, *I*^2^ = 0.0%		High	*P* = 0.421	*P* = 0.007^*^
	Adriamycins+Platinum (*n =* 1)	27/30	3.33 (0.91,12.16)	-	*P* = 0.772	-	*-*	*-*
	Adriamycins (*n =* 6)	432/417	4.08 (2.65, 6.27)^#^	*P* = 0.907, *I*^2^ = 0.0%		High	*P* = 0.260	*P* = 0.136
	Platinum (*n =* 4)	420/421	3.13 (1.64, 5.96)^#^	*P* = 0.148, *I*^2^ = 43.9%		High	*P* = 0.174	*P* = 0.028^*^
**KPS improvement (score)**^ǂ^	**Total** (*n =* **7**)	384/376	4.48 (3.18, 5.59)^#^	*P* = 0.000, *I*^2^ = 96.6%		Moderate	*P* = 0.260	*P* = 0.110
	Adriamycins+Platinum (*n =* 1)	107/104	2.32 (1.97,2.66)^#^	*-*	*P* = 0.962	-	*-*	*-*
	Adriamycins (*n =* 3)	98/93	2.62 (1.08,4.16)^#^	*P* = 0.000, *I*^2^ = 93.2%		Low	*P* = 0.602	*P* = 0.545
	Platinum (*n =* 3)	179/179	7.75 (2.55, 12.95)^#^	*P* = 0.000, *I*^2^ = 98.0%		Low	*P* = 0.602	*P* = 0.030^*^
**Child-Pugh Improvement**		275/278	3.02 (1.49, 6.10)^#^	*P* = 0.167, *I*^2^ = 40.9%	-	High	*P* = 0.308	*P* = 0.215
**Overall survival rate**	0.5-year OS (*n =* 6)	227/226	2.11 (1.34, 3.32)^#^	*P* = 0.921, *I*^2^ = 0.0%		High	*P* = 0.573	*P* = 0.696
	1-year OS (*n =* 7)	254/256	2.26 (1.56, 3.27)^#^	*P* = 0.980, *I*^2^ = 0.0%		High	*P* = 0.652	*P* = 0.840
	2-year OS (*n =* 3)	105/102	2.60 (1.36, 4.96)^#^	*P* = 0.480, *I*^2^ = 0.0%		Moderate	*P* = 0.602	*P* = 0.659
**Clinical symptoms**	Pain relief (*n =* 5)	267/260	6.98 (4.07, 11.97)^#^	*P* = 0.308, *I*^2^ = 16.7%		High	*P* = 0.221	*P* = 0.189
	Symptom score (*n =* 3)^**ǂ**^	177/161	4.36 (1.14, 7.58)^#^	*P* = 0.000, *I*^2^ = 98.7%		Moderate	*P* = 0.296	*P* = 0.015^*^
	Tumor volume reduction (*n =* 2)^**ǂ**^	71/71	4.42 (3.01, 5.83)^#^	*P* = 0.026, *I*^2^ = 79.8%		Low	*P* = 1.000	*-*
	AFP decrease value (*n =* 2)^**ǂ**^	71/71	3.07 (−0.79, 6.94)	*P* = 0.000, *I*^2^ = 97.9%		Low	*P* = 1.000	*-*
**Immunologic function**^ǂ^	CD_3_^+^ (*n =* 7)	307/297	1.10 (0.67, 1.53)^#^	*P* = 0.000, *I*^2^ = 82.1%		Moderate	*P* = 0.368	*P* = 0.059
	CD_4_^+^ (*n =* 8)	329/317	1.68 (0.84,2.51)^#^	*P* = 0.000, *I*^2^ = 94.5%		Moderate	*P* = 0.266	*P* = 0.907
	CD_8_^+^ (*n =* 6)	269/257	−0.06 (−0.67, 0.54)	*P* = 0.000, *I*^2^ = 90.3%		Moderate	*P* = 0.452	*P* = 0.826
	CD_4_^+^/CD_8_^+^ (*n =* 8)	329/317	1.19 (0.79, 1.60)^#^	*P* = 0.000, *I*^2^ = 81.0%		Moderate	*P* = 0.174	*P* = 0.360
	NK cell (*n =* 5)	253/243	2.36 (0.91, 3.82)^#^	*P* = 0.000, *I*^2^ = 97.2%		Moderate	*P* = 0.027^*^	*P* = 0.001^*^
**Liver function**^ǂ^	AST (U/l) (*n =* 4)	152/148	−1.71 (−2.75, −0.67)^#^	*P* = 0.000, *I*^2^ = 93.0%		Moderate	*P* = 0.734	*P* = 0.276
	ALT (U/l) (*n =* 8)	278/255	−1.40 (−2.14, −0.67)^#^	*P* = 0.000, *I*^2^ = 92.8%		Moderate	*P* = 0.108	*P* = 0.121
	TBIL (μmol/l) (*n =* 5)	148/138	−1.03 (−1.71, −0.34)^#^	*P* = 0.000, *I*^2^ = 86.2%		Moderate	*P* = 0.086	*P* = 0.030^*^

^**ǂ**^Standardized mean difference; ^#^Results with significant differences; ^*^Publication bias. KPS, Karnofsky Performance Status; OS, overall survival.

### Safety outcomes

Table 4 summarizes the results of toxicity associated with the different TACE arms. We found that CKI did not increase the risk of side effects, while it reduced the occurrence of nausea and vomiting, fever, hepatalgia, leukopenia, and increase in transaminase and bilirubin. Moreover, the use of TACE+CKI was safe, with mostly moderate to high-quality evidence according to GRADE assessment and moderate publication bias.

**Table 4:** Adverse events and quality of evidence

Adverse event	Subgroups/ Subscales	Participants (T/C)	OR (95% CI)	Heterogeneity (*P*, *I*^2^)	Meta-regression (*P*)	Quality of evidence	Publication bias	
							Begg’s (*P*)	Egger’s (*P*)
**Nausea/Vomiting**	**Total (*n =* 14)**	460/447	0.36 (0.27, 0.49)^#^	*P* = 1.000, *I*^2^ = 0.0%		High	*P* = 0.228	*P* = 0.117
	Adriamycins+Platinum (*n =* 2)	66/66	0.34 (0.16,0.71)^#^	*P* = 1.000, *I*^2^ = 0.0%	*P* = 0.844	Moderate	*P* = 1.000	*-*
	Adriamycins (*n =* 5)	176/167	0.42 (0.25, 0.69)^#^	*P* = 0.924, *I*^2^ = 0.0%		High	*P* = 0.142	*P* = 0.434
	Platinum (*n =* 5)	158/154	0.32 (0.19, 0.52)^#^	*P* = 0.996, *I*^2^ = 0.0%		High	*P* = 0.050	*P* = 0.134
	Other (*n =* 2)	60/60	0.38 (0.17,0.85)^#^	*P* = 1.000, *I*^2^ = 0.0%		Moderate	*P* = 1.000	*-*
**Fever**	**Total (*n =* 11)**	376/368	0.31 (0.23,0.43)^#^	*P* = 0.808, *I*^2^ = 0.0%		High	*P* = 0.213	*P* = 0.005
	Adriamycins+Platinum (*n =* 3)	105/101	0.34 (0.18,0.63)^#^	*P* = 0.452, *I*^2^ = 0.0%	*P* = 0.412	High	*P* = 0.297	*P* = 0.602
	Adriamycins (*n =* 2)	83/83	0.37 (0.13,1.08)	*P* = 0.123, *I*^2^ = 57.9%		Low	*P* = 1.000	*-*
	Platinum (*n =* 4)	128/124	0.27 (0.15,0.46)^#^	*P* = 0.867, *I*^2^ = 0.0%		High	*P* = 0.497	*P* = 0.387
	Other (*n =* 2)	105/102	0.60 (0.36, 0.96)^#^	*P* = 1.000, *I*^2^ = 0.0%		High	*P* = 1.000	*-*
**Hepatalgia**	Total (*n =* 8)	252/244	0.22 (0.14, 0.34)^#^	*P* = 0.688, *I*^2^ = 0.0%		High	*P* = 0.019^*^	*P* = 0.002^*^
	Adriamycins+Platinum (*n =* 3)	105/101	0.23 (0.12, 0.46)^#^	*P* = 0.959, *I*^2^ = 0.0%	*P* = 0.295	High	*P* = 0.297	*P* = 0.602
	Adriamycins (*n =* 1)	30/30	0.49 (0.17,1.41)	*-*		Moderate	*-*	*-*
	Platinum (*n =* 2)	57/53	0.18 (0.07, 0.44)^#^	*P* = 0.402, *I*^2^ = 0.0%		Low	*P* = 1.000	*-*
	Other (*n =* 2)	60/60	0.11 (0.03,0.33)^#^	*P* = 1.000, *I*^2^ = 0.0%		Low	*P* = 1.000	*-*
**Leukopenia**	Total (*n =* 8)	344/327	0.32 (0.22, 0.45)^#^	*P* = 0.760, *I*^2^ = 0.0%		High	*P* = 0.266	*P* = 0.415
	Adriamycins+Platinum (*n =* 1)	39/35	0.27 (0.10,0.76)^#^	*-*	*P* = 0.387	-	*-*	*-*
	Adriamycins (*n =* 3)	130/119	0.25 (0.13,0.49)^#^	*P* = 0.284, *I*^2^ = 20.5%		High	*P* = 0.602	*P* = 0.873
	Platinum (*n =* 4)	175/173	0.38 (0.23, 0.62)^#^	*P* = 0.940, *I*^2^ = 0.0%		High	*P* = 0.497	*P* = 0.400
**Increased transaminase**	Total (*n =* 11)	393/383	0.21 (0.15,0.31)^#^	*P* = 0.442, *I*^2^ = 0.0%		High	*P* = 0.087	*P* = 0.095
	Adriamycins+Platinum (*n =* 2)	66/66	0.20 (0.07,0.53)^#^	*P* = 1.000, *I*^2^ = 0.0%	*P* = 0.880	Moderate	*P* = 1.000	*-*
	Adriamycins (*n =* 5)	196/186	0.19 (0.08,0.41)^#^	*P* = 0.063, *I*^2^ = 55.3%		Moderate	*P* = 0.024	*P* = 0.374
	Platinum (*n =* 2)	71/71	0.27 (0.12,0.61)^#^	*P* = 0.600, *I*^2^ = 0.0%		Moderate	*P* = 1.000	*-*
	Other (*n =* 2)	60/60	0.14 (0.05,0.45)^#^	*P* = 1.000, *I*^2^ = 0.0%		Moderate	*P* = 1.000	*-*
**Increased bilirubin**	Total (*n =* 8)	285/283	0.27 (0.18,0.40)^#^	*P* = 0.670, *I*^2^ = 0.0%		High	*P* = 0.064	*P* = 0.036
	Adriamycins+Platinum (*n =* 2)	66/66	0.27 (0.12,0.63)^#^	*P* = 1.000, *I*^2^ = 0.0%	*P* = 0.343	Moderate	*P* = 1.000	*-*
	Adriamycins (*n =* 1)	53/53	0.50 (0.23,1.08)	*-*		-	*-*	*-*
	Platinum (*n =* 3)	106/104	0.21 (0.11,0.40)^#^	*P* = 0.444, *I*^2^ = 0.0%		High	*P* = 0.602	*P* = 0.765
	Other (*n =* 2)	60/60	0.19 (0.07,0.52)^#^	*P* = 1.000, *I*^2^ = 0.0%		Moderate	*P* = 1.000	*-*

^ǂ^Standardized mean difference; ^#^Results with significant differences; ^*^Publication bias.

## DISCUSSION

The present network meta-analysis represents the most comprehensive analysis of currently available data regarding the treatment of patients with advanced HCC with CKI combined with TACE vs TACE alone. We combined direct and indirect evidence from 44 randomized controlled trials comparing 22 different TACE arms in more than three thousand HCC patients undergoing TACE therapy to make several key observations regarding the potential efficacy and safety of CKI. First, TACE+CKI was superior to TACE-only regimens regarding overall treatment response accompanied by low heterogeneity (Figure 2). Furthermore, most of the correlations identified from subgroup analyses reached statistical significance, and heterogeneity was non-existent in most of the outcomes, with moderate to high confidence regarding the estimates (Table 2). In addition, due to the differences in treatment arms (by meta-regression), the network meta-analysis of treatment responses revealed superior efficacy for the regimens 5-FU+EPI+HCPT, THP+HCPT and 5-FU+THP+MMC+HCPT among the TACE treatment arms (Figure 4). Moreover, CKI was shown to improve the KPS score, Child-Pugh score and overall survival rate. Moreover, combination therapy with CKI could also reduce the clinical symptoms and improve immune function and liver function (Table 3). Finally, CKI did not increase the risk of adverse events but rather alleviated side effects (Table 4). Overall, compared with the TACE-only control arm, TACE+CKI was found to be both safe and efficacious for the treatment of patients with HCC.

Currently, there are a number of chemotherapeutic targeted drugs that are combined with TACE therapy to improve safety and efficacy, such as sorafenib [60–62], brivanib [63] and licartin [64], but the overall efficacy has been unsatisfactory. Additionally, the positive results reported with the multikinase inhibitor sorafenib in patients with advanced HCC need to be verified in an international cohort in the adjuvant setting. With regard to safety, the sorafenib-associated adverse events were more frequent in the combination therapy group (TACE+sorafenib). Thus, even though it may improve the treatment response and overall survival rate in patients with unresectable advanced HCC [65–66], the use of TACE+sorafenib is not recommended. Therefore, researchers have focused their attention on CKI, which can affect the immune system [67] and thus improve immune function in patients with advanced HCC, thereby enhancing therapeutic efficacy. The results can be verified in a study by Gu XB et al., in which the infusion of matrine through the hepatic artery could reduce immune function after TACE therapy and enhance the T cell immunity in the body [68–69]. In addition, in the treatment of primary HCC after surgical resection, CKI combined with TACE demonstrated a very favorable clinical efficacy, which could effectively improve the quality of life of elderly patients and prolong their survival [70]. Moreover, a study by Li showed that CKI can have a synergistic effect with TACE in killing primary HCC cells, thus alleviating the pain in the liver, improving clinical symptoms and quality of life and prolonging lifespan [71].

The current study followed the guidelines in conducting rigorous systematic reviews and network meta-analyses [72–74]. To identify as many relevant studies as possible and to decrease the risk of bias, a comprehensive search strategy was designed. Based on these considerations, we observed moderate publication bias by statistical assessment. CKI significantly increased treatment efficacy in HCC patients without increasing the incidence of adverse events. A meta-regression was performed to assess heterogeneity. Overall, the meta-regression could not identify the source of heterogeneity. In our meta-analysis, based on subgroup analyses, we obtained a negative result for only the group with a treatment duration of fewer than 10 days. The results reveal that long-term application of CKI with a standard dosage (20 ml/day) can improve therapeutic efficacy. Subgroup analyses and network meta-analyses revealed that the arms 5-FU+EPI+HCPT, THP+HCPT and 5-FU+THP+MMC+HCPT ranked best with significantly high treatment efficacies. Based on these results, we can conclude that CKI can improve the therapeutic efficacy in patients with advanced HCC undergoing TACE therapy, especially regimens with chemotherapeutic drugs with high levels of adriamycins (EPI and THP) and HCPT. This is the first study demonstrating these findings based on a meta-analysis, and there have been no other reports with similar results in the literature. Although studies on efficacy have shown a significant benefit of TACE with cisplatin or doxorubicin [10], regarding the use of chemotherapy drugs containing adriamycins (EPI and THP) and HCPT, we recommend the use of CKI as an adjuvant therapy.

Our study elaborates on the findings from primary randomization controlled trials and previous pairwise meta-analyses by systematically synthesizing efficacy and safety data [16–59]. Our meta-analysis differs from earlier studies in several ways. First, our aim was to identify the most appropriate combination of CKI and TACE, rather than simply showing that the combination is effective and safe. Second, subgroup analyses and meta-regressions were used to identify differences between the different TACE arms, CKI dosage, composition of CKI, embolizing agents and duration of treatment to determine the best options for the combination treatment. Finally, our study extends the findings from network meta-analysis and ranked the TACE treatment arms to determine the best regimen to be combined with CKI.

The network meta-analysis had some limitations that merit further discussion. First, this study was restricted to trials involving patients with advanced HCC. We excluded studies in which the patients were diagnosed with earlier stages of HCC as well as patients undergoing surgical treatment, which include a substantial number of HCC patients worldwide. In addition, the network analysis had some inconsistencies, which were mainly determined by the loop. Furthermore, positive results are more likely to be published than negative results [75]. An additional limitation of pairwise comparisons were their extensive heterogeneity (Tables 2–4), which indicated substantial variability in the outcomes reported by the included studies, even though this is often because of heterogeneity in the baseline characteristics (Table 1) and differences observed in treatment arms. Finally, there was a risk of bias. Some ways to reduce bias include defining the groups in the original studies, examining the data for each patient, and expanding the scope of the study to a global scale. In the included studies, blinding was not performed, and the quality of the studies was low (Supplementary Figure 1). The quality of most studies was only sufficient for meta-analysis.

CKI is a safe and efficacious adjuvant to TACE therapy. In addition, the use of CKI in clinical therapy is relatively new. Future trials of TACE+CKI in patients with advanced HCC should be performed on a large sample size, and their design should be robust and randomized to confirm the therapeutic efficacy and safety. Future studies should ensure that appropriate methods are used for randomization and blinding with intentions to treat. Furthermore, trials should assess outcomes using standardized or prescribed measures at similar time points. Analyses of data of each patient will be valuable for further exploration. Additional normative studies should be conducted for future network meta-analyses.

The findings of this comprehensive network meta-analysis demonstrate that combination therapy with CKI and TACE can improve treatment responses, KPS score, Child-Pugh score and overall survival rate. Moreover, combination therapy with CKI can reduce clinical symptoms and improve immune function and liver function, while reducing the risk of adverse effects. Thus, patients with advanced HCC should be encouraged to accept CKI in combination with TACE, especially with TACE regimens with high levels of adriamycins (EPI and THP) and HCPT. In patients with advanced HCC, combination therapy with CKI and TACE may be used as a first-line treatment.

## MATERIALS AND METHODS

This systematic review was reported with an previously defined protocol (PROSPERO CRD 42017073181) [76] and was performed in agreement with the PRISMA (Preferred Reporting Items for Systematic Reviews and Meta-Analyses) extension statement for systematic reviews incorporating network meta-analyses for healthcare treatments and the Cochrane Collaboration recommendations [72–74].

### Search strategy and selection criteria

We included large-scale randomized controlled trials on patients with a primary diagnosis of advanced hepatocellular carcinoma, comparing combination therapy with Compound Kushen Injection and TACE with TACE therapy alone. Studies published by August 2017 were searched in PubMed, Medline, EMBASE, and Cochrane Library, as well as four Chinese medical databases: China National Knowledge Infrastructure database, VIP database, Chinese Biomedical Literature database, and Wanfang database. We used the following search terms: “Compound Kushen Injection” OR “Matrine” and “advanced hepatocellular carcinoma” OR “advanced liver cancer” and “clinical trial” OR “randomized controlled trial”.

The inclusion criteria were as follows: randomized controlled trials in patients with a primary diagnosis of advanced hepatocellular carcinoma; patients of any age, sex, tumor stage, and Child-Pugh score; lipiodol or lipiodol+gelfoam as embolizing agents; and TACE arms with any chemotherapy drugs. We also excluded trials published only as abstract (with no additional data available from other sources). No language restrictions were implemented, and non-English papers were translated. We then screened the references in all selected articles to avoid the exclusion of relevant trials.

### Data abstraction and assessment of risk of bias

Two investigators (ZYS and HFH) independently abstracted the data on the studies, patients, and treatment-related characteristics onto a standardized form; discrepancies were resolved by consensus, by referring back to the original study or by consulting a third reviewer (ZMY or ZQC). Data on efficacy and safety were abstracted from original studies. We extracted trial design, trial size, details of treatment arms including CKI dosage and solution, chemotherapy drugs in TACE, treatment duration, embolizing agents, and type of outcome (efficacy and safety). We extracted results from intention-to-treat analyses whenever possible.

The risk of bias of the individual studies was assessed using the Cochrane risk of bias tool [77]. We assessed the following 7 items regarding risk of bias: random sequence (selection bias), allocation concealment (selection bias), blinding of participants and personnel (performance bias), blinding of outcome assessment (detection bias), incomplete outcome data (attrition bias), selective reporting (reporting bias), and other bias. All studies were classified into low risk, high risk, or unclear risk in terms of bias. Any discrepancies were resolved by consensus and arbitration by a panel of investigators within the review team.

### Outcomes

The primary efficacy outcome was treatment response. Local tumor response was measured according to the modified criteria for response evaluation in solid tumors (mRECIST) [78]; mRECIST defines the treatment response into four main categories: complete response (CR), partial response (PR), progressive disease (PD), and stable disease (SD). CR corresponds to the disappearance of intra-tumoral arterial enhancement in all target lesions, and PR corresponds to a minimum of 30% decrease in the total diameter of visible (enhancement in the arterial phase) target lesions, with reference to the total diameter of target lesions at baseline. PD is defined as an increase of at least 20% in the total diameter of viable target lesions, with reference to the smallest total diameter of viable target lesions recorded at the beginning of treatment; SD refers to the cases that do not qualify for either PR or PD.

Subgroup analyses and meta-regression were performed according to the various TACE arms (adriamycins+platinum, adriamycins and platinum), CKI dosage (20 ml/d and < 20 ml/d), composition of CKI (NS or GS, NS and GS), embolizing agents (lipiodol and lipiodol+gelfoam) and duration (≤ 10 d, 10–30 d and > 30 d). In addition, we performed a network meta-analysis according to the TACE arms (5-FU+ADM+HCPT; 5-FU+DDP; 5-FU+DDP+ADM; 5-FU+DDP+EPI; 5-FU+DDP+GEM; 5-FU+DDP+HCPT; 5-FU+DDP+MMC; 5-FU+DDP+MMC+VCR; 5-FU+EPI+HCPT; 5-FU+EPI+MMC; 5-FU+HCPT; 5-FU+MMC; 5-FU+MMC+THP+L-OHP; 5-FU+THP+MMC+HCPT; DDP+ADM+MMC; EPI; EPI+THP+L-OHP; L-OHP+GEM; THP; THP+CPT; THP+HCPT; and THP+MMC+HCPT).

The secondary efficacy outcomes were improvements in Child-Pugh score, overall survival rate, clinical symptoms, immunologic function, liver function and Karnofsky Performance Status (KPS) the KPS is considered a gold standard performance scale for cancer patients [79–80]; a significantly effective improvement in KPS score after treatment relative to that before treatment corresponds to an increase by > 20 points; an effective improvement in for KPS corresponds to an increase by 10 to 20 points; a stable KPS corresponds to an increase by < 10 points; and no change and no treatment effect is shown by a reduction in the KPS score. The safety outcomes included nausea/vomiting, fever, hepatalgia, leukopenia, increased transaminase and increased bilirubin, which were stratified by TACE arms (adriamycins+platinum, adriamycins and platinum).

### Data synthesis and statistical analysis

First, a standardized meta-analysis was performed using a random-effects model, because it is the most appropriate and conservative methodology to account for heterogeneity among trials within each comparison [81–82]. To estimate pooled odds ratios (ORs) or standardized mean differences (SMDs) with their 95% confidence intervals (CIs) incorporating heterogeneity within and between studies, STATA v 14.0 was used. Statistical heterogeneity was assessed with *P* values and the *I*^2^ statistic, with values over 50% indicating substantial heterogeneity [83]. The Begg’s and Egger’s tests were used to detect publication bias [84].

To further investigate the heterogeneity, meta-regressions and subgroup analyses were performed to assess the primary outcome data and associations according to TACE arms (adriamycins+platinum, adriamycins and platinum), CKI dosage (20 ml/d and < 20 ml/d), composition of CKI (NS or GS, NS and GS), embolizing agents (lipiodol and lipiodol+gelfoam) and treatment duration ≤ 10 d, 10–30 d and > 30 d). The *P* values in the meta-regression revealed the overall significance of the influence factors. Additionally, the *P* values were inversely proportional to the degree of heterogeneity; *P* values less than 0.10 indicate factors that could be an important source of heterogeneity.

Second, a random-effects network meta-analysis was performed using STATA v 14.0. The results of the network meta-analysis were summarized using ORs and their credible intervals (CrI) [85]. A common heterogeneity parameter was used for all comparisons, and the global heterogeneity was assessed using *P* values and the *I*^2^ statistic.

The relative efficacy and safety outcomes were derived from the combination of direct and indirect evidence obtained from network meta-analysis, which was assumed to be coherent [77]. Inconsistencies between direct and indirect sources of evidence were statistically assessed globally (by comparing the fit and parsimony of consistency and inconsistency models) and locally (by calculating the difference between the direct and indirect estimates in all closed loops in the network) [86]. When a direct connection between two treatment arms was not available, the results were based on indirect evidence.

We estimated ranking probabilities for all treatments in each TACE arm. The treatment hierarchy was summarized and reported as surface under the cumulative ranking curve (SUCRA) [87], ranging from 1, indicating that the treatment has a high likelihood of being the best, to 0, indicating that the treatment has a high likelihood of being the worst. High SUCRA score corresponds to a higher ranking of the treatment method for treatment response compared with the ranking of the other treatments.

### Quality of evidence

In addition, the quality of evidence for the primary outcomes was assessed based on the GRADE system using GRADEpro GDT [88–89]. The GRADE system assesses the risk of bias (study limitations), imprecision, inconsistency, indirectness of study results, and publication bias (classifying each as high, moderate, low, or very low) across the body of evidence to derive an overall summary of the quality of evidence.

### Patient involvement

No patients were involved in formulating the research question or assessing the outcome measures, nor were they involved in developing plans for the design or implementation of the study. None of the patients were consulted for the interpretation or compiling of the results. There is no intention to circulate the results of the research among the study participants or the relevant patient community.

## SUPPLEMENTARY MATERIALS FIGURE AND TABLE




