# Supplementary material for: Can Kushen injection combined with TACE improve therapeutic efficacy and safety in patients with advanced HCC? a systematic review and network meta-analysis
Source: Oncotarget. 2017 Sep 15;8(63):107258–72. doi: 10.18632/oncotarget.20921 (PMC5739811; doi:10.18632/oncotarget.20921)
Supplement: Supplementary file 1 [file oncotarget-08-107258-s001.pdf]

Can Kushen injection combined with TACE improve therapeutic efficacy and safety in patients with advanced HCC? a systematic review and network meta-analysis

SUPPLEMENTARY MATERIALS

Supplementary Table 1: Study characteristics. See Supplementary_Table_1

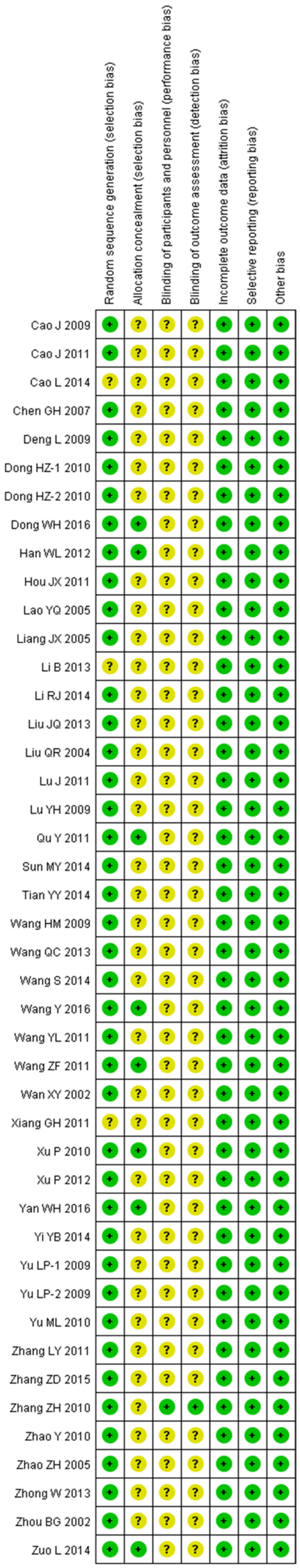

Supplementary Figure 1: Risk of bias summary.
