# Supplementary material for: Can Kushen injection combined with TACE improve therapeutic efficacy and safety in patients with advanced HCC? a systematic review and network meta-analysis
Source: Oncotarget. 2017 Sep 15;8(63):107258–72. doi: 10.18632/oncotarget.20921 (PMC5739811; doi:10.18632/oncotarget.20921)
Supplement: Supplementary file 2 [file oncotarget-08-107258-s002.doc]

**Supplementary Table 1: Study Characteristics.**

| **Publication, Year** | **Subjects** | | | | **Design** | | | | **Outcomes** |
| --- | --- | --- | --- | --- | --- | --- | --- | --- | --- |
|  | **Design** | **Participants(N,age,male%)** | **Tumor stage(I-II/III-IV)** | **Child-Pugh**  **(A/B-C)** | **TACE arm** | **TACE+CKI arm** | **Embolizing Agents** | **Duration** |  |
| Dong WH, 2016 [16] | RCT | T:108,  T+C:108,  29~81, 137/79 | - | T:52/50  T+C:55/47 | TACE :L-OHP+ GEM | TACE+ CKI: 20 ml/d, iv(NS or GS) | Lipiodol | 7-10d, ≥2 course of treatment | Treatment response, KPS improvement, CP change, immunologic function |
| Wang Y, 2016[17] | RCT | T:48, 57.4±4.3,  T+C:48,56.8±4.2, | - | T:21/27  T+C:22/26 | TACE: 5-FU+DDP+MMC | TACE+ CKI: 20 ml/d, iv(NS) | Lipiodol | 10d, 2 course of treatment | TR, AE |
| Yan WH, 2016[18] | RCT | T:38, 51. 89 ± 4. 78, 24/14  T+C:38,53.72 ±4. 77, 21/17 | - | - | TACE | TACE+ CKI: 20 ml/d, iv(NS) | - | - | TR, pain, |
| Zhang ZD, 2015[19] | RCT | T:60,  T+C:60,  55.7±6.8, 101/19 | - | - | TACE: 5-FU+HCPT+EPI | TACE+ CKI: 15 ml/d, iv(NS) | Lipiodol | 14d | pain, liver function |
| Cao L, 2014 [20] | CCT | T:42,  T+C:38, | - | - | TACE:THP+CPT | TACE+ CKI: 20 ml/d, iv(NS) | Lipiodol + Loversol | 10d | Pain, CP change |
| Li RJ, 2014 [21] | RCT | T:40,  T+C:40,  63.85, 43/37 | - | 43/27 | TACE: 5-FU+EPI+MMC | TACE+ CKI: 20 ml/d, iv(NS or GS) | Lipiodol | 2-3weeks,3 course of treatment | TR, AE |
| Sun MY, 2014[22] | RCT | T:39, 51.5, 27/12  T+C:39, 48.7, 23/16 | - | T:22/17  T+C:18/17 | TACE: 5-FU+DDP | TACE+ CKI: 20 ml/d, iv(GS) | Lipiodol | 15d,3 course of treatment | Treatment response, KPS improvement, CP change, AE,CLINICAL |
| Tian YY, 2014 [23] | RCT | T:33,  T+C:35, | - | - | TACE: 5-FU+DDP+MMC | TACE+ CKI: 20 ml/d, iv(NS) | Lipiodol | 6 weeks | TR, AE |
| Wang S,2014[24] | RCT | T:104,54.5, 94/10  T+C:107, 55.6, 91/16 | T:79/25  T+C:82/25 | - | TACE: 5-FU+DDP+EPI | TACE+ CKI: 15 ml/d, iv(NS) | Lipiodol | 14d | Pain, KPS improvement,liver function, AE |
| Yi YB, 2014[25] | RCT | T:36,52.8±7.8, 29/7  T+C:34, 48.9±11.9,22/12 | - | T:26/10  T+C:26/8 | TACE | TACE+ CKI: 20 ml/d, emulsify | Lipiodol |  | TR, OS, AE |
| Zuo L, 2014[26] | RCT | T:30,44.04±17.68, 21/9  T+C:30, 44.93±16.92,19/11 | - | - | TACE: 5-FU+DDP+ADR | TACE+ CKI: 15 ml/d, iv(GS) | Lipiodol | 14d | TR, AE |
| Li B, 2013[27] | CCT | T:101,  T+C:98,  59.7, 139/60 | - | - | TACE: 5-FU+DDP+MMC | TACE+ CKI | Lipiodol | 14d, 4 course of treatment | TR, QOL, CP, AE |
| Liu JQ, 2013[28] | RCT | T:42,58.6±1.3, 24/18  T+C:42, 58.4±1.2,25/17 | - | - | TACE: 5-FU+HCPT+EPI | TACE+ CKI: 20 ml/d, iv | Lipiodol+ Gelfoam | 2monthes | TR, KPS improvement,AE |
| Wang QC, 2013[29] | RCT | T:24,57.4, 19/5  T+C:24, 55.0,22/2 | - | T:19/5  T+C:18/6 | TACE: 5-FU+MMC+THP+ L-OHP | TACE+Matrine 0.3g, emulsify | Lipiodol | - | immunologic function |
| Zhong W, 2013[30] | RCT | T:20  T+C:20 | - | - | TACE: 5-FU+DDP+GEM | TACE+ CKI: 600mg, iv | Lipiodol+ Gelfoam | 7d | Liver function, immunologic function, AE |
| Han WL, 2012[31] | RCT | T:30,57, 18/12  T+C:30, 56,19/11 | T:21/9  T+C:22/8 | - | TACE: EPI | TACE+ CKI: 20 ml/d, iv(NS or GS) | Lipiodol | 2d, 2 course of treatment | TR, KPS improvment, CP, immunologic function ,AE |
| Xu P, 2012 [32] | RCT | T:30,  T+C:30,  53, 38/22 | - | - | TACE: THP+HCPT | TACE+ CKI: 20 ml/d, iv(NS or GS) | Lipiodol | 7-10d, ≥2 course of treatment | TR, OS, KPS improvment, immunologic function , |
| Cao J, 2011[33] | RCT | T:30,  T+C:30, | - | - | TACE: 5-FU+DDP+MMC | TACE+ CKI: 20 ml/d, iv(NS or GS) | Lipiodol | 14d, ≥2 course of treatment | TR,KPS improvment, immunologic function |
| Hou JX, 2011[34] | RCT | T:20,54, 16/4  T+C:28, 51,22/6 | - | T:13/7  T+C:21/7 | TACE: EPI+THP+ L-OHP | TACE+ CKI: 15 ml/d, iv(NS or GS) | Lipiodol | 2-3weeks | TR, liver function |
| Lu J, 2011[35] | RCT | T:35,62.8, 22/13  T+C:39, 63.4,25/14 | T:15/20  T+C:11/28 | T:15/20  T+C:16/23 | TACE: 5-FU+DDP+EPI | TACE+ CKI: 20 ml/d, iv | Lipiodol | 10d | KPS improvment, immunologic function, AE |
| Qu Y, 2011[36] | RCT | T:32,51, 19/13  T+C:32,53,21/11 | - | T:18/14  T+C:15/17 | TACE: 5-FU+DDP | TACE+ CKI: 20 ml/d, iv(GS) | Lipiodol | 14d, 2 course of treatment | TR, OS, tumor size, KPS improvment,child, AE |
| Wang YL , 2011[37] | RCT | T:23,  T+C:27,  43.2, 32/18 | - | - | TACE: 5-FU+MMC+EPI | TACE+ CKI: 20 ml/d, iv(NS or GS) | Lipiodol | 14d, 2 course of treatment | TR,KPS improvment,AE |
| Wang ZF, 2011[38] | RCT | T:31,54.5±7.67, 23/8  T+C:36, 53.5±8.32,28/8 | - | - | TACE: 5-FU+ADM+HCPT | TACE+ CKI: 20 ml/d, iv(GS) | Lipiodol | 15d, 3 course of treatment | TR,KPS improvment,AE |
| Xiang GH, 2011[39] | RCT | T:30,47, 23/7  T+C:30,46.5,25/5 | T:25/5  T+C:20/10 | - | TACE: 5-FU+THP+MMC +HCPT | TACE+ CKI: 1.2g/d, iv | Lipiodol | 4-6d, 1-7 course of treatment | TR, liver function |
| Zhang LY, 2011[40] | RCT | T:30,57.9±5.8, 19/11  T+C:30,56.4±5.6,21/9 | - | - | TACE: 5-FU+EPI+MMC | TACE+ CKI: 20 ml/d, iv(NS) | Lipiodol | 14d, 3 course of treatment | TR,AE |
| Dong HZ-1, 2010 [41] | RCT | T:33,57, 24/9  T+C:33,58.5,23/10 | T:0/33  T+C:0/33 | - | TACE: 5-FU+DDP+EPI | TACE+ CKI: 20 ml/d, iv(NS or GS) | Lipiodol | 14d, ≥2 course of treatment | TR,AE |
| Dong HZ-2, 2010[42] | RCTCT | T:33,57, 24/9  T+C:33,58.5,23/10 | T:0/33  T+C:0/33 | - | TACE: 5-FU+DDP+EPI | TACE+ CKI: 20 ml/d, iv(NS or GS) | Lipiodol | 14d, ≥2 course of treatment | TR,AE |
| Xu P, 2010[43] | RCT | T:53  T+C:53  54, 76/30 | - | - | TACE:THP+HCPT | TACE+ CKI: 20 ml/d, iv | Lipiodol | 15d, ≥2 course of treatment | TR.OS,KPS,AE |
| Yu ML, 2010[44] | RCT | T:48,53.5±5.76, 32/16  T+C:48,52.6±6.43,36/12 | - | - | TACE: 5-FU+ADM+HCPT | TACE+ CKI: 20 ml/d, iv(NS or GS) | Lipiodol+ Gelfoam | 15d, 3 course of treatment | TR,KPS,AE |
| Zhang ZH, 2010 [45] | RCT | T:38,49.3±2.74, 23/15  T+C:38,47.3±2.56,20/18 | - | T:27/11  T+C:25/13 | TACE: 5-FU+DDP+EPI | TACE+ CKI: 0.6g/d, iv( GS) | Lipiodol | 12weeks | liver function |
| Zhao Y, 2010[46] | RCT | T:30,  T+C:30,  61,36/24 | - | - | TACE: 5-FU+DDP+MMC | TACE+ CKI: 20 ml/d, iv(NS) | Lipiodol | 15d, 2-7 course of treatment | KPS, clinical symptoms, liver function |
| Cao J, 2009[47] | RCT | T:30,  T+C:30,  61,36/24 | - | - | TACE: 5-FU+DDP+MMC | TACE+ CKI: 20 ml/d, iv(NS) | Lipiodol | 15d, 2-7 course of treatment | TR, KPS, pain, immunologic function, AE |
| Deng L,2009[48] | RCT | T:20,51, 17/3  T+C:20,53,18/2 | - | - | TACE: THP | TACE+ CKI: 20 ml/d, iv | Lipiodol | 14d, 2 course of treatment | TR, pain,KPS |
| Lu YH, 2009, [49] | RCT | T:25,-, 19/6  T+C:38,-,26/12 | - | - | TACE: 5-FU+DDP+MMC+VCR | TACE+ CKI: 20 ml/d, iv(NS) | Lipiodol | 21d, 2 course of treatment | liver function, clinical symptoms |
| Wang HM, 2009 [50] | RCT | T:30,48, 26/4  T+C:27,48,24/3 | T:16/14  T+C:15/12 | - | TACE: 5-FU+DDP+ADM | TACE+ CKI: 20 ml/d, iv | Lipiodol | 10d | TR, OS, KPS |
| Yu LP-1, 2009[51] | RCT | T:30,57, 22/8  T+C:30,58,20/10 | T:16/14  T+C:5/25 | - | TACE: 5-FU+HCPT | TACE+ CKI: 20 ml/d, iv(GS) | Lipiodol | 14d, ≥2 course of treatment | TR, AE |
| Yu LP-2, 2009[52] | RCT | T:30,57, 22/8  T+C:30,58,20/10 | T:16/14  T+C:5/25 | - | TACE: 5-FU+HCPT | TACE+ CKI: 20 ml/d, iv(GS) | Lipiodol | 14d, ≥2 course of treatment | TR, AE |
| Chen GH, 2007[53] | RCT | T:40,  T+C:46,  40-711,64/24 | - | 46/40 | TACE: 5-FU+MMC+EPI | TACE+ CKI: 20 ml/d, iv(NS or GS) | Lipiodol | 2-3w, ≥3 course of treatment | TR, OS, KPS,AE, immunologic function |
| Lao YQ, 2005[54] | RCT | T:60,61, 45/15  T+C:62,58,47/15 | - | T:46/14  T+C:48/14 | TACE: THP+MMC+HCPT | TACE+ CKI: 150mg/d, iv | Lipiodol | 14d | liver function |
| Liang JX, 2005[55] | RCT | T:33,45.2, 26/7  T+C:35,44.5, 27/8 | T:16/17  T+C:18/17 | - | TACE: DDP+ADM+MMC | TACE+ CKI: 150mg/d, iv(GS) | Lipiodol | 14d | TR,clinical symptoms,liver,AE |
| Zhao ZH, 2005[56] | RCT | T:32,41.5, 23/9  T+C:30,42.5, 24/4 | - | - | TACE: 5-FU+DDP+HCPT | TACE+ CKI: 150mg/d, iv | Lipiodol | 14d | liver function, ,clinical symptoms,AE |
| Liu QR,2004, [57] | RCT | T:35,26-71, 30/5  T+C:40,29-74, 31/9 | T:23/12  T+C:24/11 | - | TACE: 5-FU +MMC | TACE+Matrine 0.6g, emulsify | Lipiodol+ Gelfoam | 3m | TR,clinical symptoms,OS,AE |
| Wan XY,2002[58] | RCT | T:39,48, 31/8  T+C:41,47.3, 32/9 | T:32/9  T+C:26/13 | - | TACE: 5-FU +MMC+CBP+ADM+HCPT | TACE+ CKI: 20 ml/d, iv(GS) | Lipiodol | 14d | clinical symptoms,AE,liver |
| Zhou BG, 2002[59] | RCT | T:20,49.5, 12/8  T+C:26,50, 17/9 | T:13/7  T+C:16/10 | - | TACE: DDP+ADM+MMC | TACE+ CKI: 16 ml/d, iv | Lipiodol+ Gelfoam | 16d, 3 course of treatment | TR,AE |

5-Fu, Fluoracil; ADM,Doxorubicin; ADM,Doxorubicin; CBP,Carboplatin; DDP, Cisplatin; EPI, Epirubicin; GEM,Gemcitabine; HCPT, Hydroxycamptothecin; L-OHP , Oxaliplatin;

MMC, Mitomycin; TACE, transarterial chemoembolization; THP , Pirarubicin;THP,Pirarubicin; VCR,vincristine.
